# An introduction to instrumental variable assumptions, validation and estimation

**DOI:** 10.1186/s12982-018-0069-7

**Published:** 2018-01-22

**Authors:** Mette Lise Lousdal

**Affiliations:** Department of Public Health, Aarhus University, Bartholins Allé 2, 8000 Aarhus C, Denmark

**Keywords:** Instrumental variable, Monotonicity, Causal inference, Unmeasured confounding, Randomization, Exchangeability

## Abstract

The instrumental variable method has been employed within economics to infer causality in the presence of unmeasured confounding. Emphasising the parallels to randomisation may increase understanding of the underlying assumptions within epidemiology. An instrument is a variable that predicts exposure, but conditional on exposure shows no independent association with the outcome. The random assignment in trials is an example of what would be expected to be an ideal instrument, but instruments can also be found in observational settings with a naturally varying phenomenon e.g. geographical variation, physical distance to facility or physician’s preference. The fourth identifying assumption has received less attention, but is essential for the generalisability of estimated effects. The instrument identifies the group of *compliers* in which exposure is pseudo-randomly assigned leading to exchangeability with regard to unmeasured confounders. Underlying assumptions can only partially be tested empirically and require subject-matter knowledge. Future studies employing instruments should carefully seek to validate all four assumptions, possibly drawing on parallels to randomisation.

## Background

Random assignment of exposure ensures that unmeasured confounding can be regarded as random [1]. By design both measured and unmeasured confounders are expected to be equally distributed across assignment groups. This leads to exchangeability i.e. if the exposure status had been reversed, the final outcome measure comparing the two groups would not have changed [2, 3]. Non-compliance may invalidate analyses based on actual received treatment if related to the risk of outcome. Employing the random assignment as an instrument may estimate the causal average effect had everyone complied [4].

In observational studies, causal inference is challenged by the lack of random exposure assignment [5]. Self-selection occurs when patients select themselves for a specific exposure. This type of confounding has been investigated within the fields of oral contraceptives, postmenopausal hormone therapy, statins and influenza vaccines and termed “compliance bias” [6], “prevention bias” [7], “healthy adherer effect” [8] and “healthy user effect/bias” [9]. The effect of preventive interventions on health outcomes may be overestimated, because those who choose to participate in general are healthier than non-participants. Confounding by indication occurs when physicians or other health professionals select patients for a specific exposure [10, 11]. Confounding by indication leads to an underestimation of the treatment effect when physicians reserve treatment for the frailest patients and an overestimation when physicians choose the healthiest patient for treatment [12]. Healthy user bias and confounding by indication are intractable biases that are difficult to rule out even after exhaustive control for prognostic [13], social and personal factors [6]. If a suitable instrument can be identified, the causal average effect among compliers may be estimated even in the presence of unmeasured confounding.

Within economics, the instrumental variable method has been commonly employed to estimate causal effects in the presence of unmeasured confounding [14]. Instruments were originally conceptualised as exogenous variables in structural equation models and assumptions related to the disturbances. For epidemiologists, the instrumental variable method and underlying assumptions may be easier conceptualised by emphasising the parallels to randomisation. The objective of this paper is to review the instrumental variable assumptions and potential validation using directed acyclic graphs and introduce the two-stage instrumental regression technique.

## Three basic assumptions

**Fig. 1:**
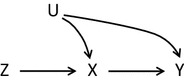
A randomised controlled trial with instrument *Z*, exposure *X*, outcome *Y* and unmeasured factors *U*

An instrument is defined as a variable that predicts the exposure, but conditional on exposure shows no independent association with the outcome. The instrument affects the outcome solely through the effect on exposure. The random assignment in a trial is an example of an ideal instrument, but a naturally occurring phenomenon may also be found in observational settings that meet the required assumptions. The underlying assumptions have been slightly differently characterised in the literature [4, 12, 14–20], but three general assumptions can be identified. Figure 1 depicts a randomised controlled trial with an assignment indicator *Z*, exposure *X* and outcome *Y* that share common causes *U*, which represents unmeasured factors that bias the association *X* → *Y*. The variable *Z* is an instrument because it meets the following three assumptions:

1. The *relevance* assumption: The instrument *Z* has a causal effect on *X.*
2. The *exclusion* restriction: *Z* affects the outcome *Y* only through *X.*
3. The *exchangeability* assumption: *Z* does not share common causes with the outcome *Y* [19]. This assumption has also been termed the *independence* assumption [15, 18], *ignorable treatment assignment* [14], or described as *no confounding for the effect of Z on Y* [16].

The *relevance* assumption is self-evident in a randomised controlled trial, where the assignment ideally determines exposure. Although assignment and treatment will not be perfectly correlated due to non-compliance, *Z* will certainly be predictive of *X*. The *exclusion* restriction is satisfied by effective double-blindness, which means that neither health professionals nor participants know the assignment [16]. Therefore, *Z* cannot have a direct impact on *Y*. Moreover, the *exchangeability* assumption is trivially satisfied because randomisation is expected to lead to equally distributed confounders across assignment groups [14].

An unbiased estimate of the average effect *X* → *Y* can be estimated from the average effects of *Z* → *Y* and *Z* → *X* [4]. The usual instrumental variable estimand for a dichotomous treatment is the ratio:

$$\frac{{E\left[ {Y|Z = 1} \right] - E[Y|Z = 0]}}{{E\left[ {X|Z = 1} \right] - E[X|Z = 0]}}$$

For a continuous treatment the instrumental variable estimand is the ratio:

$$\frac{{Cov\left( {Y,Z} \right)}}{{Cov\left( {X,Z} \right)}}$$

Intuitively, the numerator corresponds to the intention-to-treat effect of the causal effect of assignment on outcome [16, 19]. The denominator is a measure of compliance with the assigned exposure. When non-compliance increases, the denominator shrinks and inflates the diluted intention-to-treat estimate in order to estimate the causal effect had everyone complied. Applying instrumental variable methods within randomised control trials can take account of non-compliance, see for example [21, 22].

**Fig. 2:**
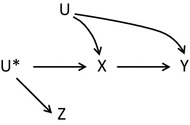
An observational study with proxy instrument *Z*, unmeasured instrument U*, exposure *X*, outcome *Y* and unmeasured factors *U*

Instrumental variable methods may be extended to observational studies if the *relevance* assumption is slightly changed to a more general version: The instrument *Z* and exposure *X* are associated either because *Z* has a causal effect on *X*, or because *X* and *Z* share a common cause [16]. In the latter instance the unmeasured causal instrument U* is the common cause of the measured surrogate or proxy instrument *Z* and the exposure *X*, see Fig. 2.

In the literature many different types of proposed instruments in observational studies can be identified such as genetic factors known as Mendelian randomisation, access to treatment based on geographic variation or physical distance to a facility, and preference for treatment based on facility or physician treatment variation [18, 19]. Some authors encourage the exploitation of natural variation [15], while others caution that the challenge of identifying a valid instrument is not trivial [16, 17]. Martens and colleagues establish a hierarchy of instruments [17], where the most valid observational instrument is a variable that is controlled by the researcher e.g. a randomised encouragement to stop smoking. Secondly, some examples of natural randomisation processes can be found e.g. Mendelian randomisation, where alleles are allocated at random in offspring. When neither an active randomisation nor a natural randomisation exists, the third opportunity is to select a source of natural variation as an instrument and carefully justify that the assumptions are satisfied. Often natural variation only gives rise to a weak association between instrument and exposure. As the degree of valid randomisation weakens, the need for careful scrutiny of the *exchangeability* assumption increases. In addition, the *exclusion* restriction must be carefully considered in the absence of blinding [17].

The three basic assumptions allow for identification of an upper and lower bound of the causal effect [4, 15, 16, 23]. Unfortunately, these bounds will typically be wide and even compatible with both a preventive effect, a causative effect or no effect at all [19]. The wide bounds underscores the uncertainty related to estimating the causal effect. Moreover, they show how much “information” that needs to be provided by a fourth assumption in order to obtain a point estimate [24].

## The fourth identifying assumption

The fourth identifying assumption is related to effect homogeneity [16, 19]. In clinical settings effects of exposure are often heterogeneous e.g. statins are more effective among patients with high levels of cholesterol than patients with low levels. Examples of homogeneous exposure effects are rare though the effect of appendectomies has been suggested as a case [12]. In the most extreme version of the homogeneity assumption, the effect of exposure *X* on outcome *Y* should be constant across individuals, which is biologically implausible. A weaker, more plausible assumption is that of no effect modification by *Z* on the *X*–*Y* causal effect in subpopulations of exposed and unexposed [19]. In other words, among the exposed the causal effect is unrelated to the instrument and likewise among the unexposed the causal effect is unrelated to the instrument. This assumption is not naturally intuitive, but it can be shown that additive effect modification by unmeasured confounders for the *X*–*Y* effect is sufficient to ensure that the assumption does not hold [19]. In practice, some of the unmeasured confounders will most likely be effect modifiers.

However, an alternative assumption that does not require effect homogeneity has been put forward. This is the assumption of *monotonicity* or *no defiers* [19, 25]. It comes at the expense of limiting the generalisability of the causal effect estimate. Imagine a simple situation with a dichotomous instrument and a dichotomous exposure. If we assume that we are capable of observing the value of the exposure under both the actual assignment and the counterfactual assignment, we can identify four different subgroups, see Table 1 [14]. In reality, only the exposure under the actual assignment is observed, and therefore we cannot distinguish between these subgroups in real life.

**Table 1:** Four subgroups defined in terms of counterfactuals by combinations of assignment and exposure

	*Z* = 0	
	*X* = 0	*X* = 1
*Z* = 1		
*X* = 0	Never takers	Defiers
*X* = 1	Compliers	Always takers

*Never takers* are the individuals that—regardless of which group they are assigned to—never would be exposed. Likewise, the *always takers* are the individuals that—regardless of assignment—always would be exposed. The *compliers* are the individuals whose exposure follows the assignment. The compliers are also referred to as the *marginal* [12] or *co*-*operative* [4] subjects. Within this subgroup the instrument is expected to achieve exchangeability. Exposure is able to follow assignment, because prognostic factors are not that weak or strong that the patient would either never get the treatment or always get the treatment. Instead treatment depends on the instrument i.e. a controlled or naturally occurring randomly varying phenomenon. For example, a new treatment that is only available at one central facility might show better outcomes for severe cases as compared to the traditional treatment available at smaller decentralised facilities. Mild cases would never be referred to the central facility, whereas severe cases would always be referred. Cases that are neither mild nor severe might be referred depending on their physical distance to the central facility. This means that when comparing two patients with similar prognostic factors, where one lives nearby and the other far away, the first might get referred to the central facility and the latter not. Had the first one lived far away and the other nearby, their exposure status would have been reversed. In this way, the instrument pseudo-randomly assigns treatment across exchangeable groups. Finally, the group of *defiers* is the individuals whose exposure is the opposite of their assignment. In the previous example this means that a patient living nearby the central facility would in fact get referred to a decentralised facility and had this patient contrary to fact lived far away, the patient would have been referred to the central facility. This group is crucial for the fourth identifying assumption, which states that there are no defiers [25].

**Fig. 3:**
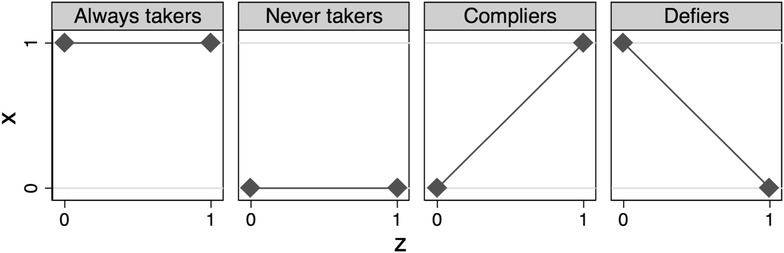
Effect of instrument *Z* on exposure *X* in four subgroups

Four simple plots in Fig. 3 clarify the connection between the naming of the *monotonicity* assumption and the concept of no defiers [19]. Always takers and never takers have a constant value of exposure regardless of assignment that is a zero causal effect of *Z* on *X*. If no defiers exist, then the only subpopulation in which *Z* can affect *X* is the compliers. This is illustrated by the monotonically increasing graph in the third plot. If no defiers exist, the effect of *Z* on *Y* will only stem from the group of compliers. Therefore the instrumental variable estimand will inflate the average causal effect to the causal effect had everyone in the population been compliers [15]. This effect estimate is termed the local average treatment effect (LATE) [14, 19, 24]. The relevance of this effect estimate has been questioned, since the group of compliers cannot be identified, and therefore it is difficult to convert the effect estimate to an estimate of practical relevance for decision makers [26]. The group of compliers as well as the effect estimate will vary from one study to another depending on the proposed instrument [12]. However, strong implausible assumptions of effect homogeneity are needed to estimate the average treatment effect in the population (ATE) [24].

## Validation of assumptions

**Fig. 4:**
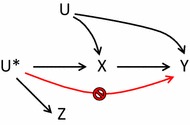
Graphical representation of *exclusion* restriction

The *relevance* assumption of a *Z*–*X* association is empirically verifiable and comprise the first step in the most common instrumental estimation technique: the two-stage least squares estimator [14–16, 19]. The first stage predicts the expected value of exposure based on the instrument. The association is evaluated using F-statistics, r^2^ or the risk difference. As a rule of thumb the instrument is declared weak if the F-statistic is less than 10 [19]. Weak instruments will result in wide confidence intervals. The *exclusion* restriction cannot be verified from the data [16, 19]. Instead subject-matter knowledge must be applied to rule out the possibility of any direct effect of the instrument on exposure, see Fig. 4. In randomised controlled trials effective double blinding ensures this. In observational studies using physician’s preference as an instrument, this assumption would be violated if the physician prescribes other drugs in combination with their preferred treatment e.g. nausea-relieving medication in combination with chemotherapy in a study evaluating side effects in different treatment regimens.

**Fig. 5:**
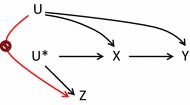
Graphical representation of *exchangeability* assumption

The *exchangeability* assumption is partially verifiable in data using measured covariates [15, 19]. A tabulation of the distribution of measured confounders across levels of the proposed instrument will reveal potential unbalances. However, confounding from unmeasured covariates cannot be ruled out. Figure 5 shows that bias may arise if *U* has a direct effect on *Z*. In controlled trials, randomisation ensures that confounders are expected to be equally distributed across assignment groups, but in observational studies special attention must be paid to proposed instruments, especially studies based on natural variation. In studies based on physical distance, another factor such as socioeconomic status that affects both treatment and outcome, may also affect distance to central facility. In preference-based studies, a clustering of high-risk patients may occur around physicians with a specific preference if patients at higher risk “doctor shop” by seeking out physicians depending on their preference [15]. Obviously, this self-assignment will violate the randomness of the instrument and create a spurious association. Although the *exclusion* restriction and *exchangeability* assumption cannot be verified from the data, different approaches to falsifying invalid instruments have been proposed [20, 27].

The fourth assumption of *monotonicity* or *no defiers* is ruled out by design in randomised controlled trials, because blinding removes the possibility of defiance [15]. In observational studies, validation requires subject-matter knowledge and is difficult to test empirically [12, 19]. When using physician’s preference as an instrument, complex decision processes with multiple factors may violate the monotonicity assumption [25]. A preference-based instrumental analysis may be supplemented with a survey of treatment plans and preferences among physicians in order to empirically assess the *monotonicity* assumption [25].

Any violations of the *exclusion* and *exchangeability* assumption will result in a biased estimate. However, a weak instrument will have a multiplicative effect on the bias in the numerator, since this is inflated by the small denominator [16, 17]. This may result in an instrumental variable estimate that is even more biased than the conventional estimate based on actual exposure. Therefore, careful consideration of possible violations is required.

## An intuitive introduction to estimation

The most common instrumental estimation technique is the two-stage least squares estimator [15, 19]. The first stage predicts the expected value of exposure based on the instrument in a linear model:

$$E\left[ {X|Z} \right] = \alpha_{0} + \alpha_{1} Z$$

The second stage then predicts the outcome as a function of the predicted exposure from the first stage:

$$E\left[ {Y|Z} \right] = \beta_{0} + \beta_{1} E[X|Z]$$

The parameter $\beta_{1}$ is equivalent to the instrumental variable estimator. Any measured covariates to predict the exposure may be added in the first stage and again in the second stage. Conditioning on these covariates will relax the assumption of marginal exchangeability to an assumption of conditional exchangeability based on the covariates [15].

**Fig. 6:**
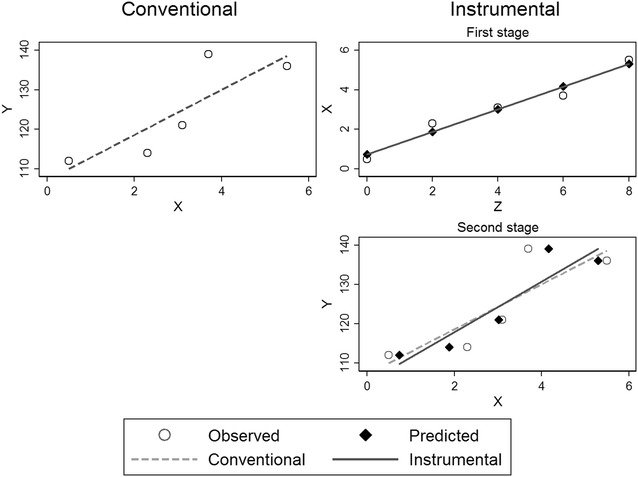
Conventional regression (left column) and two-stage instrumental regression (right column) based on hypothetical data with instrument *Z*, outcome *Y* and exposure *X*

To intuitively understand the estimation process, conventional and instrumental linear regression are presented visually in Fig. 6 based on hypothetical data. Normally, in a conventional regression the observed values of exposure constitute the independent variable that predicts the dependent variable. In the instrumental regression, the first stage shows the linear prediction of the exposure based on the instrument. In the second stage the predicted values from the above fitted line are employed as the independent variable instead of the observed values. The actual exposure has been replaced by the predicted exposure. The instrumental regression line based on predicted values shows a steeper slope than the dotted line of conventional regression that may have been affected by unmeasured confounding. The basic idea is that the predicted values are unaffected by the common unmeasured causes that confound the *X* → *Y* relation.

## Conclusions

Three basic assumptions for the instrumental variable method have been characterised in the literature, but the fourth identifying assumption of *monotonicity* has received less attention. Future studies employing instruments should carefully seek to validate all four assumptions, possibly drawing on parallels to randomisation.
